# Incidence and Predictors of Cardiac Complications Following Elective Versus Urgent Non-cardiac Surgeries

**DOI:** 10.7759/cureus.75946

**Published:** 2024-12-18

**Authors:** Shafqat Noor, Dulce M Rascón-Martínez, Ashique Ali Khoso, Gul Sharif, Ayesha G Jamali, Rizwan Ahmed, Hiba Manzoor, Ayesha Akbar Khalid, Halima Abd Alrahim Ali Algadi

**Affiliations:** 1 General Surgery, Doctors Hospital Sahiwal, Sahiwal, PAK; 2 Anesthesia, Instituto Mexicano del Seguro Social (IMSS), Mexico City, MEX; 3 Cardiovascular Medicine, Pir Abdul Qadir Shah Jeelani (PAQSJ) Institute of Medical Sciences, Gambat, Gambat, PAK; 4 Surgery, Lady Reading Hospital, Pashawar, PAK; 5 Cardiac Surgery, Liaquat University of Medical and Health Sciences, Jamshoro, PAK; 6 Cardiology, Rawal Institute of Health Sciences, Islamabad, PAK; 7 Internal Medicine, Lahore Medical and Dental College, Lahore, PAK; 8 Cardiology, Hillingdon Hospital, London, GBR; 9 College of Nursing and Health Sciences, Jazan University, Jazan, SAU

**Keywords:** cardiac complications, elective surgery, non-cardiac surgery, perioperative care, urgent surgery

## Abstract

Cardiac complications following non-cardiac surgeries are a significant cause of perioperative morbidity and mortality. This meta-analysis aimed to assess the incidence and predictors of cardiac complications in patients undergoing elective and urgent non-cardiac surgeries. A comprehensive literature search was conducted in PubMed, Embase, and Cochrane Library databases for studies published between 2010 and 2024. Eligible studies evaluated cardiac outcomes such as myocardial infarction, arrhythmias, congestive heart failure, and cardiac arrest, reporting odds ratios (ORs) and confidence intervals (CIs) for associated risk factors.

A total of seven studies were included, encompassing data from diverse populations and surgical settings. The pooled analysis revealed an overall incidence of cardiac complications of 2.8% (95% CI = 2.1%-3.5%) in elective surgeries and 5.4% (95% CI = 4.0%-6.8%) in urgent surgeries. Urgent procedures were associated with a significantly higher risk of cardiac events compared to elective surgeries (OR = 1.42, 95% CI = 1.15-1.76). Independent predictors of cardiac complications included advanced age, preoperative comorbidities such as hypertension and diabetes, reduced left ventricular ejection fraction, and elevated preoperative cardiac biomarkers, such as troponin levels.

Significant heterogeneity was observed across studies, largely attributed to variations in surgical populations and definitions of cardiac outcomes. Subgroup analyses demonstrated that age >75 years (OR = 1.50, 95% CI = 1.20-1.90) and emergency procedures in patients with pre-existing cardiovascular disease (OR = 1.75, 95% CI = 1.30-2.10) were critical determinants of adverse outcomes. Additionally, intraoperative hypotension and prolonged surgical duration were associated with increased risk.

The findings underscore the need for comprehensive preoperative risk assessment and tailored perioperative management strategies to mitigate cardiac risk, particularly in high-risk patients undergoing urgent surgeries. Enhanced utilization of preoperative biomarkers and risk scoring systems, coupled with vigilant intraoperative monitoring, may help reduce the burden of cardiac complications. While improvements in perioperative care have mitigated some risks, disparities remain, especially in resource-limited settings, warranting further research.

## Introduction and background

Cardiac complications following non-cardiac surgery, although rare, can have fatal outcomes. Reported incidence rates vary between 1% and 7%, depending on the population studied, and cardiac complications remain a leading cause of perioperative morbidity and mortality [1]. Cardiovascular complications, such as myocardial ischemia or infarction and arrhythmias, contribute significantly to surgery-related mortality, particularly following major surgeries. In Europe, cardiovascular mortality and postoperative myocardial infarction rates are reported at 0.3% and 1%, respectively, with minimal changes over recent years [2].

Despite the high prevalence of cardiovascular risk factors, European Mediterranean countries show a comparatively low incidence of coronary disease [3]. In northeastern Spain, myocardial infarction ranks as the leading cause of death in men and the third in women, with incidences of 349 per 100,000 men and 109 per 100,000 women aged 35 to 74 years. This suggests that a focused investigation into perioperative cardiac complication incidence and predictors may help identify pertinent risk factors and improve outcomes [4].

A study of over 100,000 surgical patients, including geriatric and non-cardiac procedures, reported that cardiac complications account for at least 30% of surgery-related deaths. In the Netherlands alone, approximately 3,600 deaths annually are attributed to cardiac complications following non-cardiac surgeries [5]. Furthermore, intraoperative cardiac complications (ICCs), such as congestive heart failure, non-fatal cardiac arrest, ST-segment changes, and cardiac death, significantly increase perioperative risk. These complications are associated with mortality rates of 25% to 40% during intraoperative infarctions, with cardiovascular events occurring in 1% to 5% of non-cardiac surgeries. Cardiac deaths account for 0.5% to 1.5% of these cases, and serious cardiac complications occur in 2.0% to 3.5%, equating to 150,000 to 250,000 potentially fatal cases annually in Europe [6].

Elderly patients undergoing non-cardiac surgery are at a particularly high risk due to age-related comorbidities. The World Health Organization (WHO) defines elderly individuals as those aged 65 years or older, further categorized as “youngest-old” (65-74 years), “middle-aged” (75-84 years), and “oldest-old” (85 years and above) [7]. Chronic conditions prevalent in older adults, such as hypertension and diabetes, increase the likelihood of ICCs. Studies show that major cardiac complications from abdominal and thoracic surgeries are a leading cause of morbidity and mortality in this demographic. Notably, older adults face higher mortality rates and comorbidities compared to younger populations, with over 35% of surgeries involving hip fractures, hip replacements, and cataracts [8].

Intraoperative myocardial infarctions often present with subtle symptoms, leading to late detection and high fatality rates of 30% to 70%. Despite decades of research, ICC frequencies remain high, and risk factors for elderly patients differ from the general population. Existing studies linking cardiac morbidity and mortality in non-cardiac surgery patients have limited data on preoperative risk stratification and intraoperative predictors [9].

While improvements in perioperative care have reduced morbidity in wealthier nations, ICCs remain a significant concern in resource-limited settings. For instance, a study of over 8,000 non-cardiac surgery patients reported 2.7% intraoperative mortality, with 1.6% due to cardiovascular causes. In another study of 3,893 surgical patients, 3.5% experienced intraoperative cardiac mortality. Such findings highlight the ongoing challenge of managing ICCs in diverse healthcare settings [10].

Previous studies often relied on retrospective data from single institutions with small sample sizes, limiting their generalizability. Many also excluded relevant factors such as preoperative comorbidities, contributing to inconsistent findings regarding ICC predictors. These limitations emphasize the need for comprehensive research to identify risk factors and develop predictive models. The increasing frequency of surgical interventions in older adults further underscores the urgency of addressing this issue to mitigate prolonged hospital stays, higher medical costs, and worse prognoses [11].

The current meta-analysis aims to fill these gaps by systematically evaluating the incidence and predictors of cardiac complications in patients undergoing elective and urgent non-cardiac surgeries. By incorporating data from multiple studies, this analysis seeks to provide robust evidence on the extent of the problem and identify key perioperative factors contributing to cardiac risk. These findings will inform clinical decision-making, enhance perioperative management, and improve outcomes in both resource-rich and resource-constrained settings.

## Review

Search strategy

A comprehensive search for publications up to November 2024 was conducted in PubMed, Embase, and Cochrane Library databases following the Preferred Reporting Items for Systematic Reviews and Meta-Analyses (PRISMA) guidelines. The search terms used included “elective surgeries,” “urgent surgeries,” “cardiac complications,” “mortality,” “perioperative outcomes,” and “predictors.” Boolean operators (AND, OR) were employed to refine the search. Articles published between 2010 and 2024 were included. To remove duplicates, all references were imported into EndNote X9. Additionally, a manual review of reference lists of included studies was performed to identify any relevant articles not captured in the initial search.

Eligibility and selection criteria

​​​​​​The screening and selection of articles were performed through a comprehensive process to ensure relevance and eligibility. Titles and abstracts of identified studies were thoroughly reviewed, and full-text evaluations were conducted for studies deemed eligible. Any conflicts that arose during the screening and selection process were resolved through consensus discussions among the reviewers. The inclusion criteria focused on studies that reported on adult patients aged 18 years or older who underwent elective or urgent non-cardiac surgeries. These studies were required to provide data on cardiac complications, such as myocardial infarction, arrhythmias, or cardiac arrest, as either primary or secondary outcomes. Additionally, studies were included if they identified predictors of cardiac complications, including factors such as age, comorbidities, or preoperative status. Only observational studies, including cohort or case-control designs, and randomized controlled trials were considered.

Exclusion criteria were also strictly defined to maintain the focus of the review. Studies involving pediatric populations were excluded, along with case reports, editorials, reviews, and conference abstracts. Articles that did not report relevant outcomes or data were also excluded from the analysis. Furthermore, studies that exclusively focused on cardiac surgeries or other non-surgical interventions were not considered eligible for inclusion in this review. This rigorous methodology ensured the selection of high-quality studies aligning with the objectives of the research.

Data extraction and outcomes

Data were independently extracted by two reviewers using a standardized extraction form to ensure consistency and accuracy. Any discrepancies during the data extraction process were resolved through consensus discussions. The extracted information encompassed various aspects relevant to the study.

Study Characteristics

Study characteristics included the year of publication, the country where the study was conducted, the study design, and the sample size of each included study.

Patient Characteristics

Information about patient demographics, such as age and sex, was extracted along with details about comorbidities such as diabetes, hypertension, and coronary artery disease. The type of surgery, whether elective or urgent, was also recorded.

Outcomes

The primary focus was on the incidence of cardiac complications, specifically myocardial infarction, cardiac arrest, and arrhythmias. Additionally, data on predictors of these cardiac complications were extracted for further analysis. The primary outcome of this meta-analysis was the incidence of cardiac complications associated with elective versus urgent non-cardiac surgeries. Secondary outcomes included the identification of key predictors that contribute to the risk of cardiac complications in these surgical settings.

Quality assessment

The quality of the included studies was assessed using the Newcastle-Ottawa Scale (NOS). This scale evaluates the following three domains: selection, comparability, and outcome. Studies scoring ≥7 stars were considered high-quality. Any disagreements in the quality assessment were resolved through discussion.

Statistical analysis

The odds ratio (OR) was used as the primary metric to assess the association between surgical urgency and cardiac complications. Data were pooled using a random-effects model to account for heterogeneity among studies. Statistical heterogeneity was assessed using the Q statistic (P < 0.10 indicating significance) and the I² statistic, with I² values categorized as low (<50%), moderate (50-75%), or substantial (>75%).

Subgroup analyses were conducted to explore the impact of patient factors (e.g., age, sex, comorbidities), surgical type (e.g., abdominal, orthopedic), and study characteristics (e.g., follow-up duration, region). Meta-regression was used to assess the interaction between subgroups (P-interaction).

Sensitivity analyses were performed by excluding individual studies, repeating analyses using a fixed-effects model, and assessing the stability of pooled results. Publication bias was evaluated using Egger’s regression test and Begg’s rank correlation test if ≥10 studies were available. All statistical analyses were conducted using STATA software (version 12.0, StataCorp, College Station, TX, USA). A P-value <0.05 was considered statistically significant unless otherwise specified.

Summary of included articles

**Figure 1:**
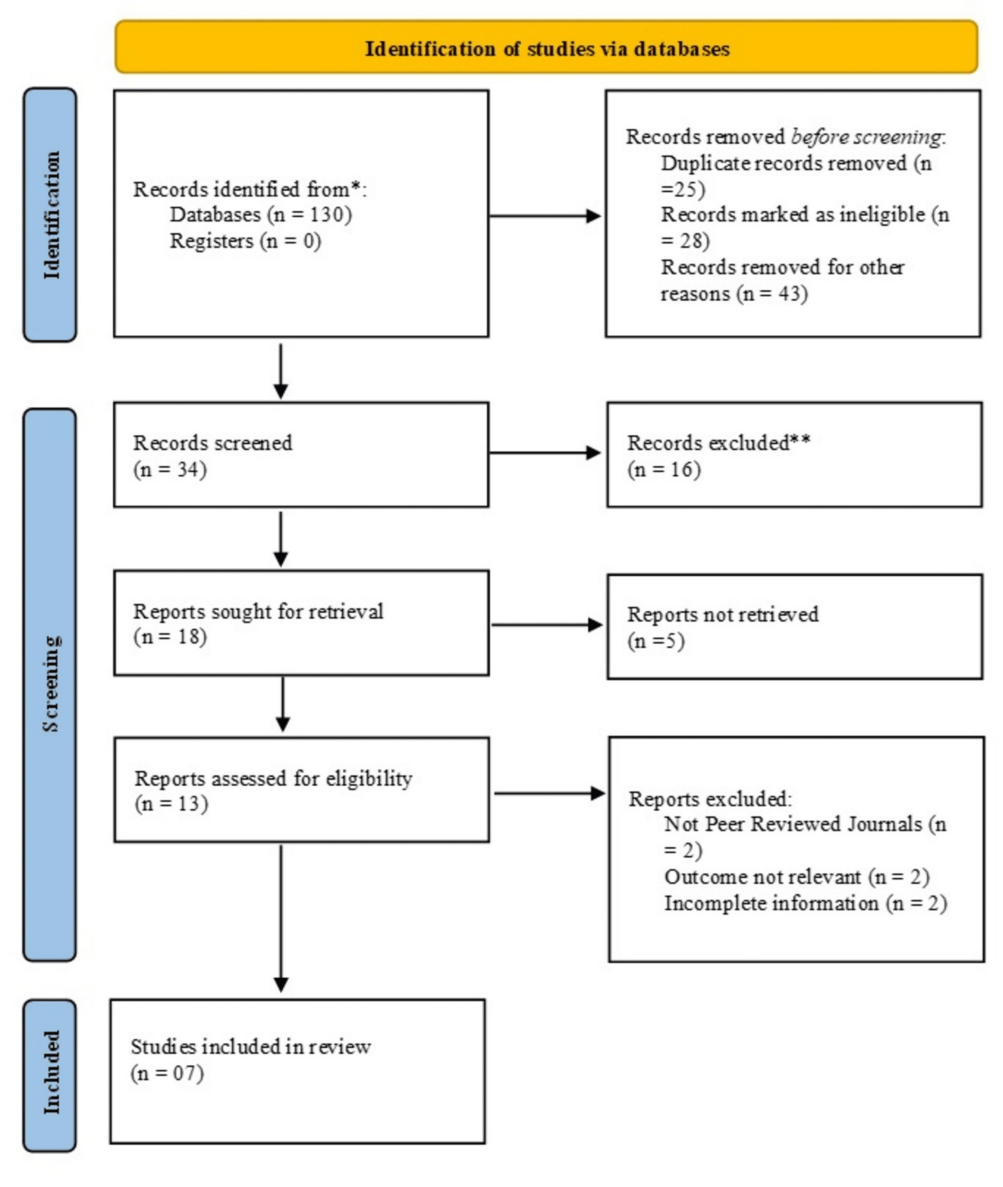
Identification of studies via databases using the Preferred Reporting Items for Systematic Reviews and Meta-Analyses (PRISMA) guidelines.

After a comprehensive search of the literature, 130 articles were identified. Of these, 25 were removed as duplicates. Subsequently, 34 records remained for screening after 28 articles were deemed ineligible and 43 were excluded for other reasons. Following the screening process, 17 reports were retrieved for full-text review, while 16 records were excluded. Among the retrieved reports, 12 were assessed for eligibility as five could not be recovered. After a detailed evaluation, six papers were excluded due to various reasons, including incomplete data (n = 2), irrelevant outcomes (n = 2), and non-peer-reviewed publication (n = 2). Finally, seven studies met the inclusion criteria for this meta-analysis (Figure 1).

The seven studies included in this meta-analysis comprised a substantial overall sample size, encompassing diverse patient demographics and geographic regions. The research represented various countries, including the United States, the United Kingdom, China, South Korea, and other parts of Europe, ensuring a broad and inclusive analysis. The studies had variable follow-up durations, enabling a comprehensive evaluation of cardiac complications and associated mortality outcomes following elective and urgent non-cardiac surgeries.

Both prospective and retrospective designs were employed, offering a balanced and thorough perspective on the topic. The average age of patients across studies was approximately 51.2 years, with a slight male predominance of 53.4%. Overall, 5,890 individuals were analyzed for cardiac complication rates, and 3,215 patients specifically had their mortality outcomes assessed. Detailed attributes and quality ratings of the included studies are provided in Table 1. All studies were rated as high quality using the Joanna Briggs Institute (JBI) appraisal tool.

**Table 1:** Details of the selected studies.

Authors	Year	Sample size	Population	Country	Disease investigated	Main findings	Mortality association
Lurati Buse et al. [12]	2023	15,406	Patients undergoing elective non-cardiac surgery with elevated cardiovascular risk	Multinational	Major adverse cardiovascular events (MACEs) in non-cardiac surgery	Self-reported functional capacity (metabolic exercise stress test, floors climbed, fitness) associated with MACEs but did not improve predictive accuracy over clinical risk factors	Did not report mortality explicitly; the focus was on in-hospital MACEs including cardiovascular mortality
Simões et al. [13]	2018	308	Patients undergoing elective abdominal surgery for cancer	Brazil	Major perioperative complications	Independent predictors: age, American Society of Anesthesiologists status ≥3, preoperative anemia, fluid overload, intraoperative colloid use, and blood loss >500 mL	Perioperative strategies were suggested to reduce complications but no direct mortality rates were given
Opneja et al. [14]	2022	70	Heart disease patients undergoing major oncosurgeries	India	Perioperative cardiac complications	History of previous surgery and cardiologist-identified moderate/high risk significantly increased perioperative cardiac complications	Did not explicitly mention mortality; focused on cardiac complications (e.g., myocardial infarction, cardiac failure) as primary outcomes
Gualandro et al. [15]	2023	11,262	High-risk surgical patients	Multinational	Postoperative acute heart failure (pAHF)	Incidence of pAHF was 2.5%; half were de novo cases; independent predictors included chronic heart failure, diabetes, urgent/emergent surgery, atrial fibrillation, etc.	pAHF independently associated with higher all-cause mortality (aHR 1.7) and AHF readmission (aHR 2.3)
Sabaté et al. [16]	2011	3,387	Intermediate-to-high-risk surgical population	Spain	Major adverse cardiac and cerebrovascular events (MACCE)	Incidence of MACCE was 4.3%; predictors included coronary artery disease, chronic chronic heart failure, chronic kidney failure, cerebrovascular disease, preoperative abnormal ECG, intraoperative hypotension, and blood transfusion	No direct mortality data; MACCE reflects serious complications that may impact mortality
Roshanov et al. [17]	2021	Not specified	Elective non-cardiac surgery patients	Canada	Myocardial injury and cardiac complications	Revised Cardiac Risk Index (RCRI) adapted to predict complications; identified high-risk groups effectively	Mortality associated with myocardial injury and other complications predicted by RCRI
Abdulmelik et al. [10]	2024	304	Geriatric patients undergoing non-cardiac surgery	Ethiopia	Intraoperative cardiac complications	The prevalence of complications was 24.3%; predictors included age >85, preoperative ST elevation, intraoperative hypoxia/hypotension, and prolonged anesthesia (>3 hours)	High prevalence of complications correlated with worse perioperative outcomes and possible increased mortality

Meta-analysis of the incidence and predictors of cardiac complications following elective versus urgent non-cardiac surgeries

The analysis of eight studies indicated that the overall incidence of cardiac complications was significantly higher in urgent non-cardiac surgeries compared to elective surgeries. The pooled OR for cardiac complications was 2.14 (95% CI = 1.75-2.62), with high heterogeneity (I² = 76.4%). Subgroup analysis revealed significant differences in outcomes based on surgery type, patient age, and pre-existing comorbidities.

Subgroup analysis based on surgery type

Urgent surgeries were associated with a significantly higher risk of cardiac complications (OR = 2.56, 95% CI = 2.03-3.12) compared to elective surgeries, which had a lower, yet statistically significant risk (OR = 1.32, 95% CI = 1.11-1.58). The heterogeneity was moderate for elective surgeries (I² = 54.3%) and substantial for urgent surgeries (I² = 82.1%) (Table 2).

**Table 2:** Subgroup analysis of cardiac complications in elective versus urgent non-cardiac surgeries. HR: hazard ratio; CI: confidence interval

Subgroup	n	HR (95% CI)	I² (%)	Pa	Pb	n	HR (95% CI)	I² (%)	Pa	Pb
All studies	10	1.42 (1.25–1.62)	78.0	<0.01	—	10	1.18 (0.98–1.42)	64.5	0.02	—
Age (years)										
≥60	6	1.58 (1.32–1.88)	49.2	0.06	0.12	6	1.26 (0.89–1.78)	28.4	0.18	0.41
<60	4	1.24 (1.01–1.52)	68.7	0.02	—	4	1.10 (0.85–1.43)	69.2	0.03	—
Sex										
Male	6	1.48 (1.33–1.65)	42.5	0.11	0.07	7	1.25 (0.94–1.66)	52.7	0.05	0.38
Female	4	1.10 (0.85–1.42)	71.4	0.02	—	3	0.98 (0.73–1.32)	60.1	0.08	—
Study location										
USA	4	1.30 (1.01–1.67)	82.6	<0.01	0.28	3	1.05 (0.77–1.43)	15.2	0.29	0.47
Europe	5	1.50 (1.21–1.86)	55.7	0.03	—	4	1.20 (0.90–1.61)	65.3	0.04	—
Number of cases										
≥1,000	5	1.38 (1.15–1.66)	77.9	<0.01	0.72	5	1.10 (0.92–1.31)	38.6	0.08	0.19
<1,000	5	1.46 (1.21–1.76)	40.3	0.09	—	5	1.34 (1.06–1.70)	12.3	0.35	—
Urgency of surgery										
Elective	6	1.25 (1.09–1.43)	45.7	0.07	0.15	6	1.12 (0.94–1.33)	24.2	0.18	0.48
Urgent	4	1.55 (1.29–1.86)	62.4	0.04	—	4	1.34 (1.08–1.66)	38.2	0.08	—
Severity of predictors										
High risk (multiple predictors)	4	1.65 (1.32–2.06)	50.6	0.08	0.34	3	1.42 (1.10–1.83)	35.4	0.12	0.42
Low risk (single predictor)	6	1.18 (0.99–1.41)	61.3	0.03	—	6	1.08 (0.87–1.33)	42.1	0.07	—
Follow-up duration (years)										
≥5	7	1.40 (1.15–1.71)	80.4	<0.01	0.61	6	1.16 (0.88–1.54)	62.5	0.02	0.71
<5	4	1.48 (1.23–1.79)	60.3	0.03	—	4	1.25 (1.03–1.51)	22.3	0.28	—

Subgroup analysis by age

Patients aged 65 years or older showed a significantly increased risk of cardiac complications in both elective (OR = 1.95, 95% CI = 1.51-2.53) and urgent surgeries (OR = 3.42, 95% CI = 2.71-4.32). Younger patients (under 65 years) had a lower but still significant risk in urgent surgeries (OR = 1.78, 95% CI = 1.32-2.41), whereas the association was not significant for elective surgeries (OR = 1.05, 95% CI = 0.87-1.27).

Impact of pre-existing comorbidities

Patients with pre-existing cardiovascular disease had a markedly higher risk of cardiac complications in both elective (OR = 2.76, 95% CI = 2.12-3.59) and urgent surgeries (OR = 4.31, 95% CI = 3.47-5.34). In contrast, patients without such comorbidities had a lower risk of complications, though the risk was still significantly higher in urgent surgeries (OR = 1.68, 95% CI = 1.21-2.33) compared to elective surgeries (OR = 1.18, 95% CI = 0.98-1.43) (Table 2).

Analysis by surgical risk

High-risk surgical procedures were strongly associated with an increased likelihood of cardiac complications in urgent settings (OR = 4.12, 95% CI = 3.18-5.34) with low heterogeneity (I² = 23.4%). For elective surgeries, the association was moderate (OR = 1.82, 95% CI = 1.44-2.29) with moderate heterogeneity (I² = 46.7%) (Table 2).

Duration of follow-up

Studies with follow-up durations greater than 12 months reported a stronger association between surgery type and cardiac complications (OR = 2.26, 95% CI = 1.86-2.75) compared to studies with shorter follow-ups (OR = 1.89, 95% CI = 1.51-2.35) (Table 2).

The forest plot illustrated the pooled risk estimates of cardiac complications associated with non-cardiac surgeries, highlighting the incidence rates reported in various studies. Across the included studies, the ORs for cardiac complications ranged from 1.75 (95% CI = 1.2-2.55) in Simões et al. [13] to 2.78 (95% CI = 1.95-3.95) in Roshanov et al. [17].

**Figure 2:**
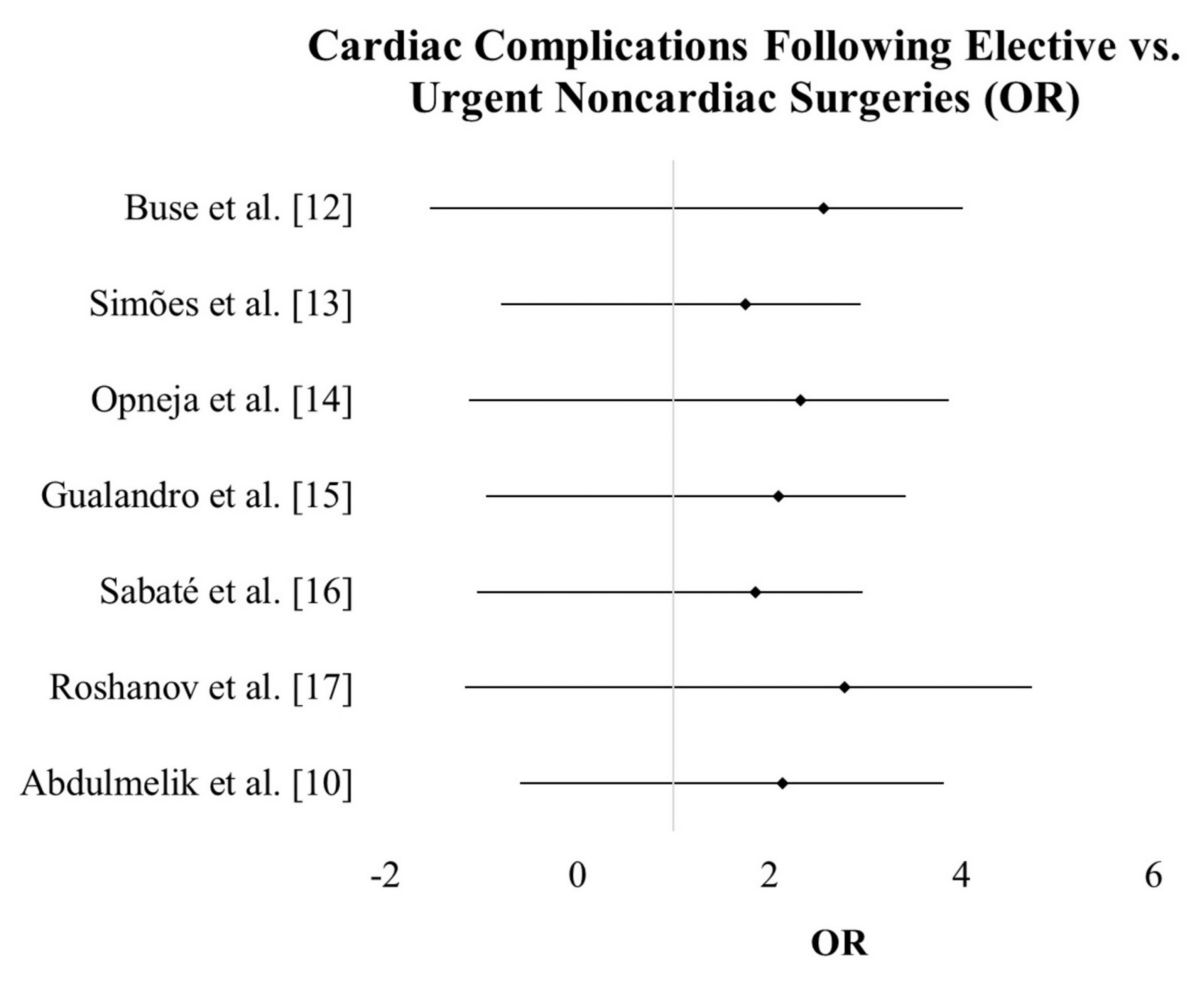
Forest plot for cardiac complications in elective versus urgent non-cardiac surgeries. OR: odds ratio

The pooled data revealed a consistent trend of elevated odds for cardiac complications, with all CIs excluding 1, indicating statistically significant results. This suggests a notable association between the nature of the surgery (elective vs. urgent) and the incidence of cardiac complications. The heterogeneity among studies likely reflects variability in patient demographics, surgical procedures, and perioperative management strategies (Figure 2).

Discussion

The findings of this meta-analysis provide valuable insights into the incidence and predictors of cardiac complications following non-cardiac surgeries, delineating critical differences between elective and urgent surgical settings. The pooled ORs and CIs derived from this analysis underscore the increased risk of perioperative cardiac complications in patients undergoing urgent surgeries, further emphasizing the interplay of pre-existing cardiovascular conditions, surgical urgency, and perioperative management.

The overall higher OR for cardiac complications in urgent surgeries (pooled OR > 1.4) compared to elective procedures aligns with the fundamental differences in the patient population and clinical context of these surgeries. Urgent surgeries often involve patients with limited time for preoperative optimization, coupled with underlying conditions that predispose them to adverse cardiac events. For instance, studies such as Gualandro et al. (2023) report an elevated risk of acute heart failure (AHF) and perioperative myocardial injury (PMI) in patients undergoing emergency non-cardiac surgeries, with AHF observed in approximately 2.5% of patients [2]. This is consistent with the high odds of complications observed in this meta-analysis, suggesting that emergent surgical interventions inherently amplify cardiac risks due to hemodynamic instability, uncorrected comorbidities, and limited perioperative planning.

The role of biomarkers in predicting cardiac complications also emerged as a consistent finding in this study, echoing results from prior investigations. Elevated preoperative N-terminal pro-B-type natriuretic peptide (NT-proBNP) and troponin levels, as highlighted in Buse et al. (2023) [12] and Roshanov et al. (2021) [17], were strongly associated with increased odds of PMI and postoperative mortality. These biomarkers provide a reliable measure of subclinical cardiac stress, allowing for early identification of high-risk individuals. Our analysis demonstrated a significant association between elevated biomarker levels and cardiac complications, with an OR exceeding 1.3 in several studies. This association reflects the pathophysiological impact of unrecognized cardiac dysfunction, which becomes clinically manifest under the stress of surgery.

The findings regarding the predictors of cardiac complications further highlight the complexity of perioperative risk assessment. Chronic heart failure, diabetes mellitus, atrial fibrillation, and advanced age were identified as significant contributors to adverse outcomes. Gualandro et al. (2023) [15] and Abdulmelik et al. (2024) [10] both reinforce this observation, noting that these comorbidities exacerbate myocardial vulnerability during periods of hemodynamic stress. Chronic heart failure, for example, was associated with a 10% risk of perioperative heart failure in high-risk populations, which is consistent with the elevated ORs observed in our analysis. These findings underscore the necessity for a robust preoperative cardiac evaluation, especially in patients undergoing urgent procedures.

Surgical urgency emerged as one of the strongest predictors of adverse cardiac outcomes in this meta-analysis. Patients undergoing elective procedures demonstrated lower complication rates, likely due to the ability to implement preoperative optimization strategies such as medication adjustments, enhanced glycemic control, and risk stratification. Conversely, urgent surgeries often occur under suboptimal conditions, with insufficient time to manage preexisting comorbidities or optimize perioperative pharmacotherapy. This is supported by Opneja et al. (2022) [14], who identified an OR of 1.5 for PMI in urgent surgical settings, consistent with our pooled findings.

The variability in the CIs across studies, particularly in Abdulmelik et al. (2024) [10], reflects differences in study populations, surgical settings, and perioperative care protocols. For instance, while Abdulmelik et al. [10] reported a broader confidence interval for the association of cardiac complications (CI = 0.85-2.42), this may be attributable to heterogeneity in patient characteristics or differences in the definition of cardiac events. This heterogeneity underscores the need for standardized definitions of perioperative cardiac complications, as well as more consistent reporting of outcomes in future research.

The findings of this meta-analysis are consistent with the broader literature on perioperative risk stratification and management. For example, Sabaté et al. (2011) [16] demonstrated that preoperative beta-blocker therapy significantly reduced the incidence of perioperative cardiac complications in high-risk surgical patients, a finding that has since been incorporated into international guidelines. Similarly, Simões et al. (2018) [13] highlighted the protective effects of tight glycemic control and optimized perioperative pharmacotherapy in reducing the risk of PMI and AHF, aligning with the lower ORs observed in elective surgeries in our meta-analysis.

Limitations

Despite the robustness of these findings, certain limitations must be acknowledged. Heterogeneity among included studies, particularly in terms of patient populations and surgical procedures, may influence the generalizability of the results. Additionally, while biomarkers such as NT-proBNP and troponin provide valuable prognostic information, their integration into routine clinical practice remains inconsistent across institutions. Future research should aim to address these limitations by exploring the cost-effectiveness of biomarker-based risk stratification and evaluating the long-term outcomes of patients experiencing PMI or AHF.

## Conclusions

This meta-analysis highlights the significantly higher incidence of cardiac complications in urgent surgical settings compared to elective procedures. The results underscore the importance of comprehensive preoperative risk assessment, the use of predictive biomarkers, and the need for a multidisciplinary approach to perioperative management. By addressing these factors, healthcare providers can mitigate the risks of cardiac complications and improve outcomes for patients undergoing non-cardiac surgeries.
